# Selective degradation of ribosomes during oncogene-induced senescence: molecular insights and biological perspectives

**DOI:** 10.1080/15548627.2024.2319022

**Published:** 2024-02-21

**Authors:** Aida Rodríguez López, Lisa B. Frankel

**Affiliations:** a Danish Cancer Institute, Copenhagen, Denmark; b Biotech Research and Innovation Centre, University of Copenhagen, Copenhagen, Denmark

**Keywords:** Oncogene-induced senescence, ribosomes, selective autophagy, translation, ubiquitination, USP10

## Abstract

Ribosomes are conserved macromolecular machines that are responsible for protein synthesis in all cells. While our knowledge of ribosome biogenesis and function has increased significantly in recent years, little is known about how ribosomes are degraded under specific cellular conditions. We recently uncovered that ribosomes are efficiently turned over by selective macroautophagy/autophagy during oncogene-induced senescence (OIS). By profiling the ribosome interactome in human fibroblasts undergoing OIS, we discovered a key role for the de-ubiquitinating enzyme USP10 in guiding this process. Release of USP10 from ribosomes during senescence leads to their enhanced ubiquitination and selective sequestering by autophagy through the SQSTM1/p62 receptor protein. This process is important for sustaining senescence-associated metabolome and secretome alterations.

Senescence plays a significant role in various biological processes, ranging from embryonic development to tissue remodeling, and is strongly associated with the development of disease, including cancer and other aging-related pathologies. A key characteristic of this multifaceted process is a state of cell cycle arrest, which can be initiated by diverse stimuli. Oncogene-induced senescence (OIS) is triggered in response to aberrant oncogenic signaling, resulting in a sustained proliferative arrest that can act as an effective barrier against tumorigenesis. The complexity of this response is underlined by the fact that OIS also promotes tumor development and progression. For instance, the persistent production of a unique pro-inflammatory secretome, known as the senescence-associated secretory phenotype (SASP), can exert pro-tumorigenic effects through its interplay with the tumor microenvironment.

Despite a complete growth arrest, senescent cells remain metabolically active and undergo significant remodeling. Autophagy plays an important role in this regard, by regulating protein and organelle homeostasis in a bulk and selective manner. In our recent study, we demonstrate how ribosomes, the crucial interpreters of genetic information, are selectively targeted and degraded by autophagy during OIS [1]. While ribosomes exhibit remarkably long half-lives in unstressed conditions, we show that human fibroblasts undergoing OIS display a high rate of ribosome turnover through autophagy. Using the pH-sensitive fluorescent mKeima reporter, fused to both large and small subunit ribosomal proteins, we observed by live-cell imaging, flow cytometry and western blotting, that ribosomes are efficiently delivered to lysosomes upon induction of OIS. Inhibition of autophagy, either chemically or genetically, clearly abrogates ribophagic flux, confirming the autophagy-dependency of this observation. Supporting these results, transmission electron microscopy revealed an accumulation of endogenous ribosomes within autophagosomes of OIS cells.

Because our data suggested that this process is regulated independently of bulk autophagy, this prompted our search for specific molecular signals governing ribophagic flux in senescence. We conducted ribosome co-immunoprecipitations of both small and large subunits followed by mass spectrometry and identified several alterations in the ribosome-associated proteome of senescent relative to proliferating cells. Among these, we found USP10 (ubiquitin specific peptidase 10) to be depleted from the ribosome interactome of OIS cells. Assessing this in further detail by polysome profiling, we confirmed a loss of USP10 across individual subunits, monosomes and polysomes, which coincides with an enhanced global ubiquitination, particularly on the small subunit. Among small subunit proteins, RPS2 displays a distinct high-molecular weight form, solely detectable in senescent conditions. We confirmed the sensitivity of this site to USP10, suggesting USP10’s direct impact on the altered ubiquitination status of senescent ribosomes. Interestingly, reenforcing the USP10-ribosome interaction in senescent cells significantly decreases ribosome ubiquitination and, consequently, ribophagic flux of both subunits, revealing USP10 as a key player in the regulation of ribosome homeostasis. We identified lysine 275 in RPS2 as a functionally important ribosome “eat-me” signal, as the mutation of this lysine to an arginine (K275R) reduces degradation of both subunits. However, this finding does not exclude the potential contribution of additional sites that remain to be uncovered. Through analogous mechanisms, ubiquitin plays an important role during the selective clearance of other autophagy substrates, including mitochondria, ER, protein aggregates, peroxisomes and pathogens with important implications for cellular health.

During selective autophagy, designated receptors can bridge between the membrane-anchored Atg8-family proteins and the selected cargo. In this study, we identified for the first time, the selective autophagy receptor protein SQSTM1, as a ribosome receptor. Interestingly, we found that whereas SQSTM1 binds to the ribosome in proliferating cells, this interaction is significantly enhanced upon induction of senescence. Importantly, depletion of SQSTM1 strongly abrogates the autophagy-mediated turnover of both ribosomal subunits. Our data additionally suggest that SQSTM1 preferentially binds to ubiquitinated ribosomes, as its affinity for the ubiquitination-defective RPS2 mutant is decreased. A potential direct binding site for SQSTM1 on the ribosome remains unknown and although these results point to SQSTM1’s importance, this does not exclude potential contributions from other selective autophagy receptors yet to be identified.

We did not detect major alterations in global protein synthesis or total ribosome levels in OIS cells, suggesting that decay of ribosomes may be counterbalanced by a continuous new production to uphold the translational capacity. Hence it is possible that OIS-associated ribophagy may serve as a mechanism for qualitative, rather than quantitative, ribosome sorting. In line with this suggestion, it has recently become evident that ribosome specialization, through a variety of modifications, may serve to tune translation dynamics during cellular state transitions. Interestingly, we found that ribophagy-compromised senescent cells exhibit a reduced secretion of SASP components, which could not be explained by altered transcription of SASP mRNAs. Hence, selective ribosome degradation may assist in rewiring or tuning translation according to altered cellular needs during OIS. Since we observe a more prominent ubiquitination on individual subunits and monosomes, relative to actively translating polysomes, ribophagy may also serve to optimize translation by degrading low-efficiency ribosomes during OIS. Collectively, our findings lead us to propose a model, in which selective ubiquitination of ribosomes guided by USP10 dynamics can contribute to the cellular sorting of ribosome pools through autophagy, with potential impact on translational tuning during phenotypic switches (Figure 1).

**Figure 1.**
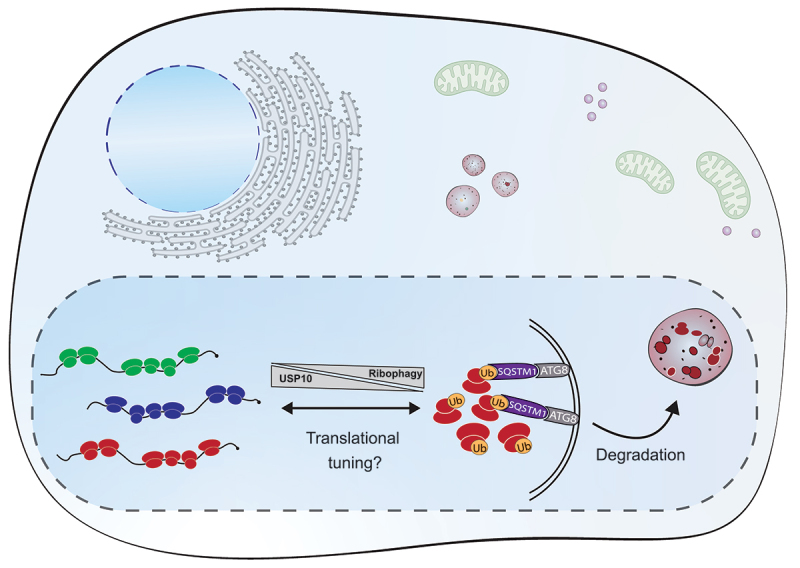
USP10 regulates ribosome dynamics during oncogene-induced senescence. USP10 dissociation from ribosomes leads to their enhanced ubiquitination and subsequent recognition by the selective autophagy cargo receptor SQSTM1. Ribosomes are subsequently encapsulated within autophagosomes and degraded in lysosomes. The selective degradation of ribosomes may serve as a mechanism for sorting ribosome pools and adjusting translation according to altered cellular needs during cell fate transitions. ATG8, mammalian Atg8-family protein.

Our comparative metabolomics analysis from ribophagy-competent and -incompetent cells suggested that ribosome turnover fuels the generation of RNA- and protein-derived metabolites through lysosomal breakdown. Considering that a major fraction of total cellular RNA (>80%) is contained within mammalian ribosomes, we hypothesized that ribophagy may serve to replenish RNA-derived metabolites. In line with this, we found that ribophagy, but not bulk autophagy, is highly sensitive to intracellular nucleoside availability. Yet we still lack information regarding the upstream signaling cues guiding this pathway, including the triggers of USP10 dissociation from the ribosome as well as the E3 ligases regulating this process. Beyond the identified lysine in RPS2, it is likely that additional ubiquitination sites or other types of ribosomal modification, can earmark ribosomes for degradation. Further investigations will help to uncover potential differences in translational characteristics of degraded and non-degraded ribosome pools and may provide a better understanding of how cellular state transitions are initiated and maintained. Considering the broad implications of senescence in tissue remodeling, cellular differentiation and aging-related pathologies including cancer, this knowledge can contribute with new understanding of fundamental cellular processes and inform future therapeutic directions.
